# Potential drivers of microbial community structure and function in Arctic spring snow

**DOI:** 10.3389/fmicb.2014.00413

**Published:** 2014-08-07

**Authors:** Lorrie Maccario, Timothy M. Vogel, Catherine Larose

**Affiliations:** CNRS UMR 5005, Environmental Microbial Genomics, Laboratoire Ampère, École Centrale de Lyon, Université de LyonEcully, France

**Keywords:** Arctic, snowpack, cryosphere, metagenomic, microbial adaptation

## Abstract

The Arctic seasonal snowpack can extend at times over a third of the Earth’s land surface. This chemically dynamic environment interacts constantly with different environmental compartments such as atmosphere, soil and meltwater, and thus, strongly influences the entire biosphere. However, the microbial community associated with this habitat remains poorly understood. Our objective was to investigate the functional capacities, diversity and dynamics of the microorganisms in snow and to test the hypothesis that their functional signature reflects the snow environment. We applied a metagenomic approach to nine snow samples taken over 2 months during the spring season. *Fungi*, *Bacteroidetes,* and *Proteobacteria* were predominant in metagenomic datasets and changes in community structure were apparent throughout the field season. Functional data that strongly correlated with chemical parameters like mercury or nitrogen species supported that this variation could be explained by fluctuations in environmental conditions. Through inter-environmental comparisons we examined potential drivers of snowpack microbial community functioning. Known cold adaptations were detected in all compared environments without any apparent differences in their relative abundance, implying that adaptive mechanisms related to environmental factors other than temperature may play a role in defining the snow microbial community. Photochemical reactions and oxidative stress seem to be decisive parameters in structuring microbial communities inside Arctic snowpacks.

## INTRODUCTION

The cryosphere is defined as the portion of Earth where the water is in solid form (Miteva, 2008). It includes sea ice, freshwater ice, glaciers, ice sheets, permafrost, and snow cover. Snow, which can cover over one third of the terrestrial surface (Miteva, 2008), influences global energy and moisture budget and, thereby, influences climate (Hinkler et al., 2008). Snow is also an interface between different biosphere compartments such as soil, aquifers, sea ice, and the atmosphere (Pomeroy and Brun, 2001; Dominé and Shepson, 2002; Schimel et al., 2004; Schmidt and Lipson, 2004). While the snowpack appears to be a critical component of the cryosphere, it is disappearing. The snow cover was estimated to be reduced by 17.8% in the Northern Hemisphere from 1979 to 2011 (Derksen and Brown, 2013) and this reduction has influenced climate regulation and snow-covered ecosystems. However, the concept of the snowpack as an ecosystem itself remains largely unexplored and its ecological role has probably been underestimated (Larose et al., 2013a).

Snowpack is formed by the accumulation of deposited ice crystals that encountered post-depositional modifications (Domine et al., 2008). In this cold porous media, microorganisms are subjected to environment-specific physical and chemical conditions, such as low nutrient concentrations, desiccation, freeze-thaw cycles, solar irradiation and therefore highly reactive photochemistry during summer and darkness during winter, in addition to low temperatures. Therefore, snow was not considered suitable to support life but only to trap microorganisms in a vegetative state before releasing them to other environments upon melting (Cowan and Tow, 2004), but this view is being challenged. Arctic snow microorganisms have been partially characterized using various culture dependent and independent approaches. Recent molecular approaches were based on the extraction of DNA from snow samples and the identification of specific functional genes, such as ribosomal genes (coding16S rRNA), and genes involved in mercury (Hg) resistance or nitrogen cycling (Larose et al., 2010a, 2013b; Møller et al., 2011). Some potential active members of the microbial community were identified in Antarctic snow by sequencing cDNA retrotranscribed from extracted 16S rRNA (Lopatina et al., 2013). These studies demonstrated that diverse microorganisms are present and potentially active in the snow with variable cell density and may be involved in various biological processes. However, details concerning activity and metabolic capabilities of snow microbial community remain limited.

If the Arctic snowpack is a functional ecosystem, then the microbial community inhabiting it should have functional genomic signatures related to their adaptation to the specific conditions specific of this environment. Several physiological adaptations have been described for microorganisms surviving under cold conditions based on psychrophilic microbial isolates (Cloutier et al., 1992; Kumar et al., 2002; Groudieva et al., 2004; Gilbert et al., 2005). Although the described increased membrane fluidity and synthesis of cold adapted enzymes are critical to life in the cold, other physical and chemical parameters might be equally critical in the Arctic snowpack. For example, photon-induced radiation is also a recognized cause of extreme conditions (Rothschild and Mancinelli, 2001). These photochemical reactions and the associated oxidative capacity have been described as playing a major role in snowpack chemistry (Grannas et al., 2007), but the impact on snow microbial community remains unknown. Our objective was to investigate the functional capacities, diversity and dynamics of the microorganisms in snow and to test the hypothesis that their functional signature reflects the snow environment. Our approach was to compare the annotated functional DNA sequences in the microbial community to other communities and to known gene families associated with different stresses such as oxidative stress in relation to high UV irradiance.

## MATERIALS AND METHODS

### SAMPLING PROCEDURE

**FIGURE 1:**
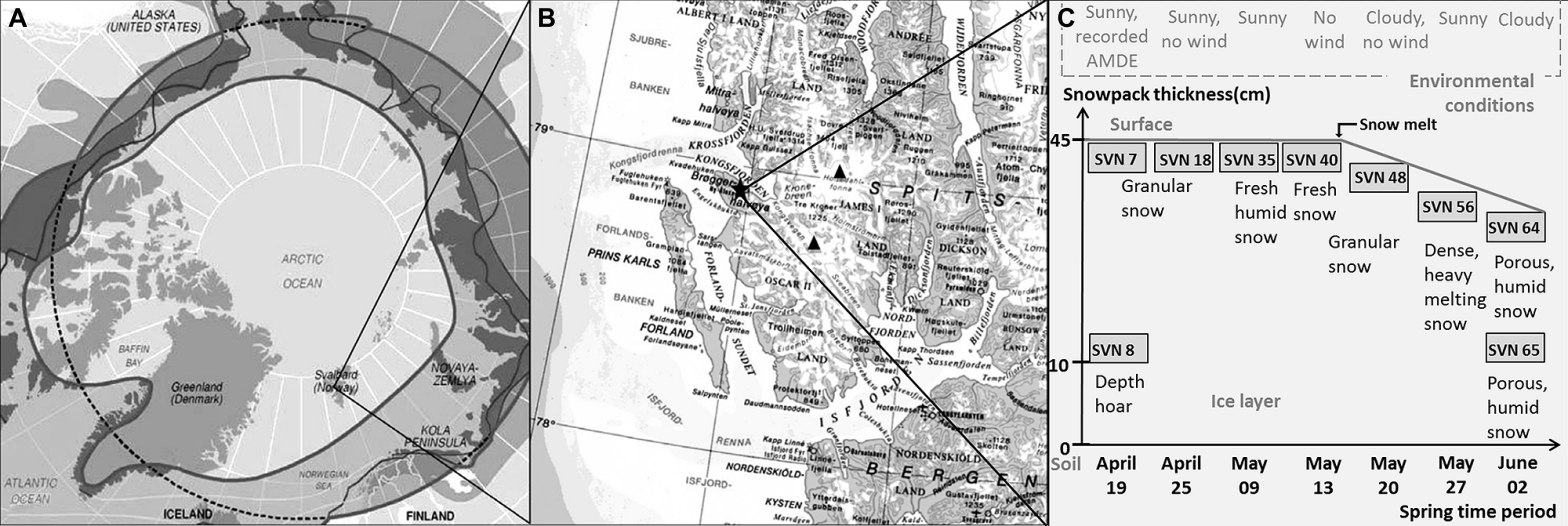
**Sampling site and procedure description – (A) Svalbard Archipelago (Norway) – **(B)** Sampling site along the south coast of the Kongsfjorden in Ny-Ålesund – **(C)** Samples description (AMDE, atmospheric mercury deposition event; scheme modified from Larose et al., 2010b)**.

Sampling site and procedure is illustrated in **Figure 1**. Samples were taken during a 2008 springtime field campaign in Ny-Ålesund (Svalbard, Norway, 78°56′N, 11°52′E). Shallow pits (total snow pack depth of 45 cm at the beginning of the field season, snow melt from mid-May) were dug between April and June at the same sampling site with a 50 m^2^ perimeter with restricted access located along the south coast of the Kongsfjorden (please consult Larose et al., 2013c for a complete description of the samples). Surface (3 first cm) and basal snow samples (10 cm above the ground) were collected in three 3 L sterile sampling bags using a sterilized Teflon shovel. To avoid contaminating the snow, Tyvex^®^ body suits and latex gloves were worn during sampling and gloves were worn during all subsequent sample handling. The nine samples chosen in this study for metagenomic analyses were representative of eight distinct groups defined by chemical and taxonomical analysis (Larose et al., 2013c). Snow chemistry was analyzed as described previously (Larose et al., 2010b). Briefly, total Hg was measured with a Tekran Model 2600 using USEPA method 1631 revision E and bioavailable Hg was determined using a *mer-lux* biosensor at the field laboratory. Samples for methylmercury and chemical analysis were shipped frozen to the laboratory in France where methylmercury was analyzed by purge and cryotrapping gas chromatography and inorganic ions and organic acids were measured by suppressed ion chromatography using a Dionex ICS 300. Chemical data are provided in **Table 1**.

**Table 1:** Snow samples chemistry.

Sample Number	SVN7	SVN8	SVN18	SVN35	SVN40	SVN48	SVN56	SVN64	SVN65
pH	6.4	4.9	5.5	4.7	5.1	4.0	4.2	5.1	6.4
Mercury (ng L^-1^)	40.7	1.9	58.5	1.2	3.7	7.7	0.8	1.1	3.3
MeHg (pg L^-1^)	BDL	BDL	BDL	BDL	BDL	BDL	0.5	BDL	0.2
BioHg (ng L^-1^)	6.7	1.6	7.3	2.1	1.5	1.2	1.1	NA	NA
MSA (μmol L^-1^)	BDL	BDL	BDL	1.0	1.4	1.1	1.6	BDL	0.6
Chloride (μmol L^-1^)	21456.5	156.1	623.3	107.8	30.7	37.4	51.4	54.9	1456.7
Natrium (μmol L^-1^)	19410.9	141.8	545.1	92.5	30.4	28.6	42	50.2	1258.2
Bromide (μmol L^-1^)	47.0	0.3	0.7	0.2	0.1	0.1	0.1	0.1	4.0
Sulfate (μmol L^-1^)	2105.6	11.6	64.2	9.6	38.8	13.7	11.4	0.5	76.4
Ammonium (μmol L^-1^)	50.7	1.3	4.4	3.6	6.9	4.3	7.2	1.4	14.2
Potassium (μmol L^-1^)	394.5	2.5	11.7	1.8	1.0	0.6	1.0	0.4	24.0
Magnesium (μmol L^-1^)	4341.5	32.0	129.5	20.5	6.8	5.5	11.2	3.5	276.1
Calcium (μmol L^-1^)	828.5	7.1	45.9	13.4	9.8	10.4	7.7	6.5	88.6
Nitrate (μmol L^-1^)	13.3	2.2	4	4.1	5.3	6.2	8.4	BDL	1.9
Nitrite (μmol L^-1^)	BDL	0.2	0.3	BDL	BDL	BDL	0.2	BDL	BDL
AGly (μmol L^-1^)	0.2	0.4	0.5	0.5	0.7	0.4	0.5	0.3	2.6

*MeHG, methyl mercury; BioHG, bioavailable mercury (Schroeder et al., 1998); MSA, methyl sulfonic acid; Agly, acetate-glycolate; NA, not available; BDL, below detection limit).*

### MICROBIAL SAMPLE PROCESSING

Samples were processed immediately after collection in the field laboratory. Samples were left to melt at room temperature prior to being filtered onto sterile 0.22 μM 47 mm filters (Merck Millipore, Germany) using a sterile filtration unit (Nalge Nunc International Corporation, USA) and filters were stored in sterile bead-beating tubes at -20°C until further analysis. Procedural blanks were carried out by filtering Nanopure water (Siemens, Germany) using the same procedure.

### DNA EXTRACTION

DNA was extracted using the protocol outlined in Larose et al. (2013c). Briefly, filters were chopped and placed in a Fastprep^®^ bead-beating tube (Lysing matrix E, MP Biomedicals, USA) to which 1 mL of DNA extraction buffer and 20 mg mL^-1^ lysing enzyme (*Trichoderma harzianum*, Sigma L1412) were added. Tubes were left at room temperature for 1 h and then frozen at -20°C overnight. The frozen tubes were incubated at 65°C for 30 min and placed in a Fastprep^®^ bead-beater (MP Biomedicals, USA) set at speed 5.5 for 30 s. DNA was extracted from the water phase with an equal volume of chloroform:isoamyl alcohol (24:1) and precipitated with isopropanol.

### PYROSEQUENCING

DNA extracted from environmental samples were sequenced by GATC (Constanz, Germany) using a Roche 454 Titanium pyrosequencer. Since the required DNA yield for pyrosequencing was 2 μg/50 μL, which could represent up to 1200 L of snow (DNA yield between 1.6 and 16 ng per L of snow), the DNA extracted from each sample was amplified using multiple displacement amplification with the illustra^TM^ GenomiPhi^TM^ HS DNA Amplification Kit (GE Healthcare, USA). Amplification was carried out according to the manufacturer’s instructions and purified by addition of 3.5 volumes of both RA1 and 70% ethanol followed by centrifugation on Nucleospin Tissue XS columns. Further washing was carried out according to the manufacturer’s instructions (Nucleospin, Machery-Nagel, Germany). No amplification was obtained using extractions carried out on field blanks.

### SEQUENCE ANALYSIS

The fasta sequences obtained were filtered for errors using cd-hit, blasted against the NCBI-NR database using the BLASTX default settings (Altschul et al., 1990; Benson et al., 2005) and analyzed using MEGAN4 (Huson et al., 2011). In parallel, metagenomic datasets were analyzed using the Metagenome Rapid Annotation with Subsystem Technology (MG-RAST; Miteva, 2008). Reads were taxonomically and functionally annotated by similarity searching against SEED database (Overbeek et al., 2005) with a maximum *e*-value of 10^-5^. Annotated data were analyzed at a broad taxonomical level (Phylum/classes) and at the second level of hierarchical functional subsystems classification from SEED database including 477 subsystems representing the collection of functional roles that make up a metabolic pathway, complex, or a class of proteins. We compared the different snow samples taken during springtime for the global community composition. The relative abundance of reads in the fifty most represented functional subsystems in the snow datasets were tested for their correlation with chemical parameters (pH, Hg, methylmercury, etc., see **Table 1**). The resulting Pearson correlation matrix was then visualized in a heatmap from the R-Package “gplot” (Ihaka and Gentleman, 1996). We compared functional subsystem distributions from snow metagenomes with other metagenomes from different ecosystems publically available on the MG-RAST platform (listed in Table S1; Miteva, 2008; DeAngelis et al., 2011; Delmont et al., 2011b; Varin et al., 2012a). Read distributions among the different functional subsystems for each ecosystem were then analyzed with the statistical software STAMP (Parks and Beiko, 2010) using analysis of variance as the statistical test parameter. The relative distributions of annotated reads in functional subsystems between ecosystems were also analyzed by principal component analysis (PCA).

## RESULTS

### SNOW METAGENOMES CHARACTERISTICS

Snow metagenomic datasets harbored on average 27,000 sequences with a length of 330 nucleotides. The smallest and the biggest datasets were obtained from the samples SVN65 (12,181 sequences) and SVN7 (42,989 sequences), respectively. Taxonomic annotation efficiency at a broad taxonomic level (phylum/classes) was high; with a proportion of unannotated reads varying between 5 and 12% of the total sequences. However, functional annotation efficiency was low; the percentage of reads with no occurrence with genes with known functions in the database varied between 60 and 88% (for SVN8 and SVN40, respectively). Detailed relative abundances of each functional or taxonomical group are directly available on MG-RAST software under the accession number indicated in Table S2. The raw metagenomic dataset can be also downloaded from the MG-RAST website.

### FUNCTIONAL AND TAXONOMICAL DYNAMIC OF SNOWPACK MICROBIAL COMMUNITY

**FIGURE 2:**
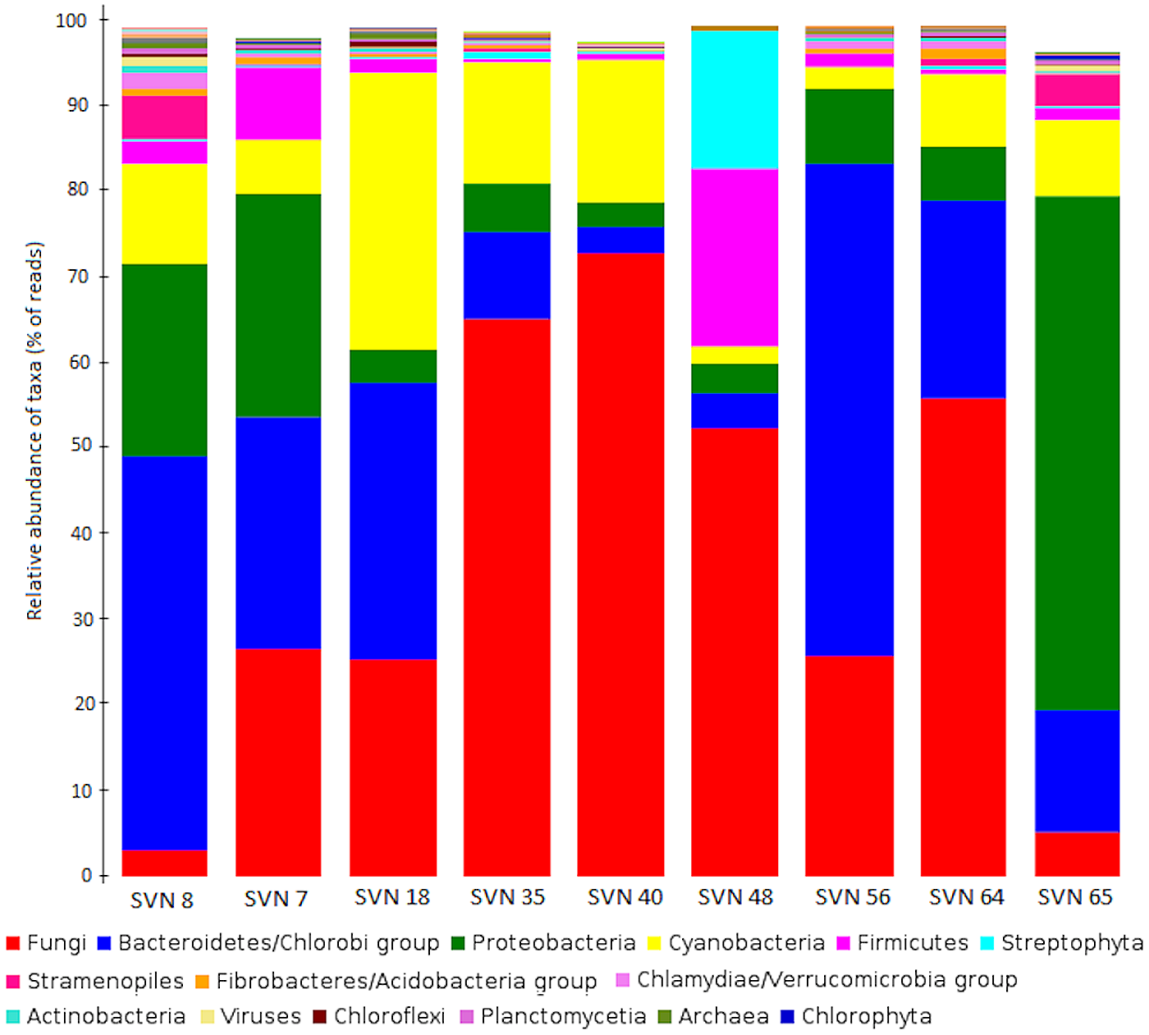
**Comparison of the major phyla/classes (NCBI-Taxonomy in MEGAN) in all snow samples.** Data is plotted as the percentage of sequence reads annotated to genomes within each phyla/class. The legend is classified in decreasing order of read numbers.

*Fungi* represented the taxon with highest amount of annotated sequence reads, followed by *Bacteria* with major phyla *Bacteriodetes/Chlorobi* (37%) *and Proteobacteria* (34%) (**Figure 2**). *Cyanobacteria* (*Nostocales*, *Chroococcales*) represented approximately 10% of the classes, except for in one surface snow sample from May 20th (SVN48). *Archaea* domain had the least amount of annotated reads and was only detected in the early season snow samples (SVN7, SVN8, SVN18). Some reads were similar to genomic sequences of species characterized as psychrophile or psychrotolerant with their highest relative abundance in basal samples and during late spring (sample SVN65). We observed variability in community structure from samples throughout the field season. Reads related to *Fungi* were dominant in surface snow metagenomes sampled between the 25th of April (SVN18) and the 27th of May (SVN56) and reached up to 70% of annotated reads in the sequenced sample from the 13th of May (SVN40). We also observed differences in community composition between surface and basal snow (SVN8 and SVN65), where reads annotated to bacteria from *Proteobacteria*, *Bacteroidetes*/*Chlorobi,* and *Cyanobacteria* were dominant relative to* Fungi* that were in relatively low annotated read abundance.

**FIGURE 3:**
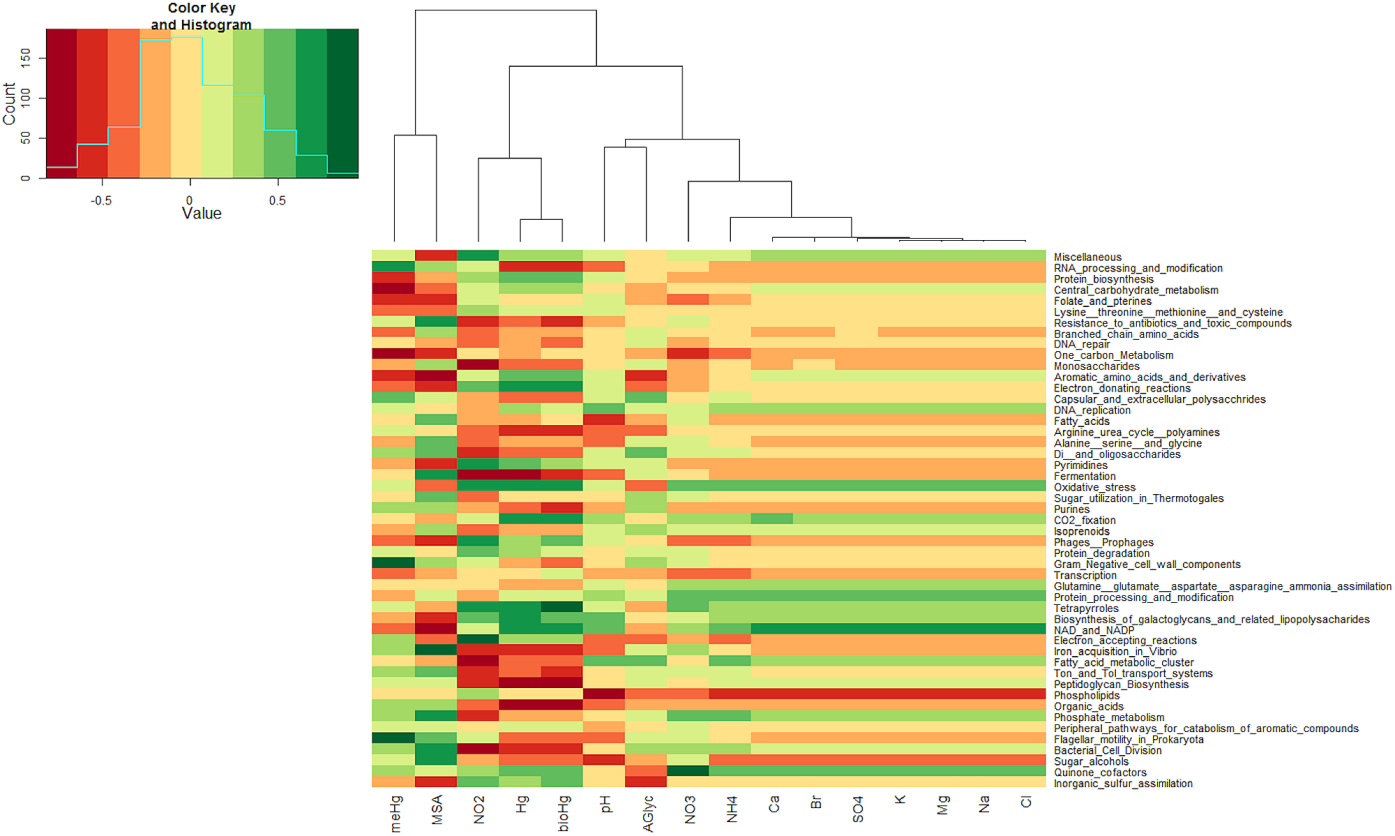
**Heatmap from Pearson correlations between chemical data and the first 50 (in abundance) subsystems at the second level of seed classification.** Functional subsystems are ordered from the most to the least abundant.

Sequence reads from the snow metagenomes were classified into metabolic functions using the SEED database Of the annotated reads, most were classified as carbohydrate metabolism genes (10–19%), followed by virulence, amino acid, protein, DNA, cell wall, cofactors, and respiration. The functional profile varied among snow samples. For example, the proportions of reads associated with virulence varied between 8.72% for the surface snow sample svn35 to up to 18.10% for the surface snow sample svn56 sampled 3 weeks later. Among virulence associated reads, the majority corresponded to antibiotic and toxic compound resistance, and pollutant biodegradation and reach up to 91% of the annotated reads in sequences from sample svn56. The chemical parameters measured in snow samples also varied between samples; surface versus basal samples and during the Spring season (**Table 1**). Detailed analyses of these abiotic data have been published in a previous article (Larose et al., 2010b). Based on the heatmap from the Pearson correlation matrix (**Figure 3**), many of these physico-chemical factors correlated with the functional annotation from the high throughput sequencing. For example, total Hg, bio-available Hg (bio-Hg) concentrations were correlated to oxidative stress, tetrapyrroles (Cobalamin and coenzyme B12 biosynthesis and Heme/siroheme biosynthesis) and NAD/NADP metabolism. MSA was also positively correlated to iron acquisition and quinine cofactors.

### FUNCTIONAL SIGNATURE OF SNOWPACK MICROBIAL COMMUNITY

**FIGURE 4:**
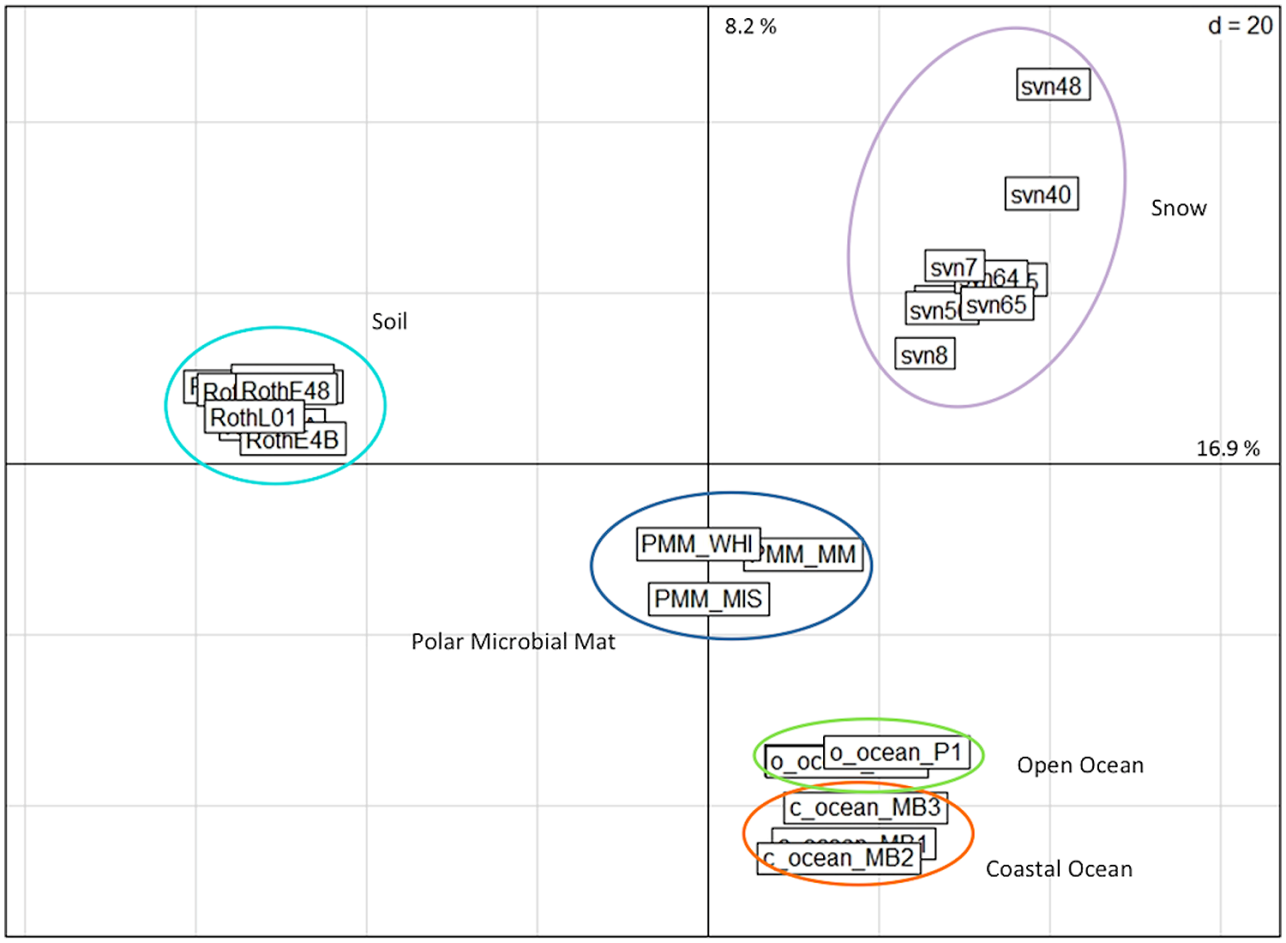
**PCA based on the relative distribution of annotated sequences (*E*-value < 10^-5^) classified in different functional subsystems by MG-Rast software. Distributions were normalized as a function of the number of annotated sequences for each metagenome.** The snow samples were compared to metagenomes from different ecosystems. PMM, polar microbial mats Arctic and Antarctic (Varin et al., 2012a,b); Roth, Rothamsted soil (Delmont et al., 2011a,b); PR, Puerto Rico soil (DeAngelis et al., 2010); O-Ocean, open ocean (Delong et al.; Giovannoni et al.); C-ocean, coastal ocean (Delong et al.).

The relative abundance of annotated reads of functional subsystems was compared among different ecosystem metagenomic datasets referenced in Table S2: snowpack, polar microbial mats for samples corresponding to the cryosphere and soil (forest and grassland) together with oceans (coastal and open oceans) for mesophilic environments. All of the snow samples grouped together in the PCA of the functional read distributions and were separated from the other ecosystems (**Figure 4**). However, different snow samples from the same sampling site in Svalbard and from the same sampling season were more dispersed than samples collected for other environmental groups even those that included sequences from different sampling sites, time periods, extraction protocols, and sequencing technologies.

**FIGURE 5:**
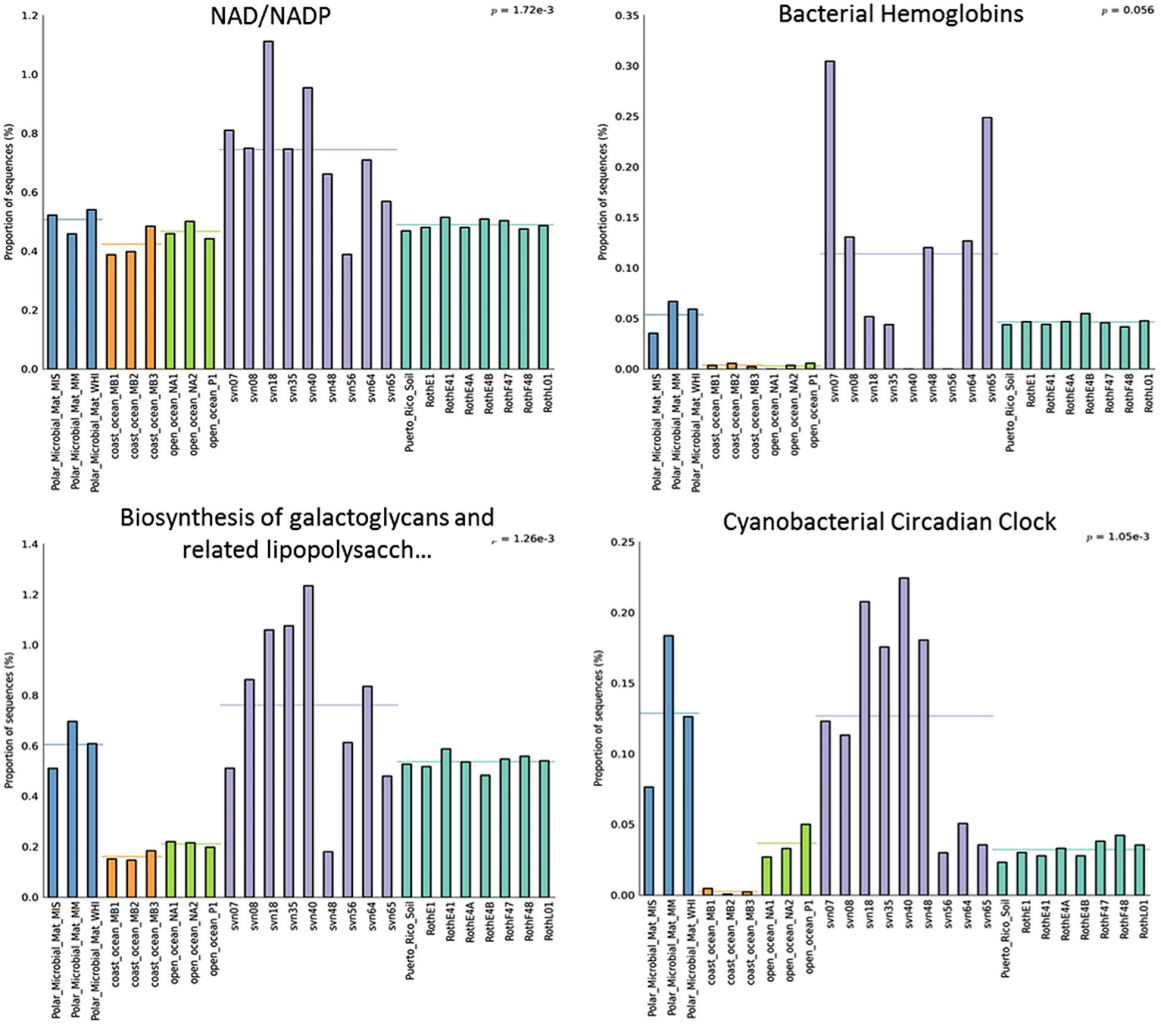
**Relative distribution (in percentage of annotated sequences) of different functional subsystems (annotation with MG-Rast software).** Distributions between different ecosystems were compared using multiple group analyses (ANOVA, Tukey–Kramer) in STAMP. PMM, polar microbial mats Arctic and Antarctic (Varin et al., 2012a,b), Roth, Rothamsted soil (Delmont et al., 2011a,b), PR, Puerto Rico soil (DeAngelis et al., 2010); O-Ocean, open ocean (Delong et al.; Giovannoni et al.), C-ocean, coastal ocean (Delong et al.).

Several functional subsystems were more represented in terms of normalized read numbers in the snowpack than in sequences from other environments. All subsystems at the second level of seed classification that were more abundant in snow samples are listed in Table S2. As an example, we focused on four different subsystems, illustrated in **Figure 5**. For NAD/NADP metabolism, the proportion of reads represents on average 0.8% of annotated sequences and was significantly higher in snow samples (*p*-value 1.72 × 10^-3^). Proportions of reads associated with biosynthesis of galactoglycans and associated polysaccharides were also globally more elevated in early spring snow samples (up to 1.2%). In addition, the percentage of reads related to cyanobacterial circadian clock were significantly different among environments (*p*-value 1.05 × 10^-3^) with a greater representation in polar microbial mat and snow samples (0.13% of sequences on average for both). Bacterial hemoglobin associated reads also seem to be more represented in most snow samples despite the non-significant *p*-value 0.056 which is likely explained by snow sample heterogeneity. Among all functional subsystems, we also focused on those associated to cold-resistance mechanisms, whose distributions in the different environments are provided in Table S3. Although the associated genes were found in our snow samples, most of them were not statistically more represented in cryospheric environments (both snow and polar microbial mat) than in other environments. However, genes related to fatty acid desaturases and biosynthesis of galactoglycans that are involved in cold resistance were relatively more dominant in our snow samples and polar mats than in other ecosystems.

## DISCUSSION

Snow is increasingly considered to be a diverse and active ecosystem, but the microbial community inhabiting this environment remains poorly understood (Larose et al., 2013a). High throughput sequencing was used to determine the composition and potential functional capability of the microbial community in the snow. We sequenced nine snow samples taken over a Spring sampling season. The resulting metagenomic datasets were analyzed for both their taxonomic and their functional profiles based on comparing the sequences to known microorganisms and proteins.

Bacterial community composition of our snow samples had been analyzed in detail previously using a 16S rRNA gene phylogenetic microarray (Larose et al., 2013c). Despite differences inherent to the methodologies used, the bacterial taxa detected were similar with both techniques (microarray and high throughput sequencing) and consistent with previous studies on snow in the Arctic and Antarctic (Carpenter et al., 2000; Amato et al., 2007; Larose et al., 2010a; Lopatina et al., 2013; Møller et al., 2013). Bacterial sequences retrieved from our snow metagenomes were mostly annotated to *Proteobacteria* and *Bacteroidetes* and include reads affiliated to known psychrophiles or psychrotolerant bacteria. Total microbial community sequencing increased our taxonomic description of snow communities and highlighted the abundance of eukaryotic representatives that constituted, in some of the snow metagenomes, a majority of the total reads (up to 70% in SVN40). Most of these sequences were annotated to *Fungi*, mainly *Ascomyceta*, which has representatives described in other cold environments like Canadian High Arctic snowpack and Arctic Ice (Gunde-Cimerman et al., 2003; Harding et al., 2011). As short and highly conserved domains can lead to unspecific taxonomic assignment of metagenomics reads (Huson et al., 2007), we analyzed these data at broader taxonomical levels (phylum/classes). To our knowledge, this is the first study to include prokaryotic and eukaryotic representatives together to produce a global picture of snow microbial community structure and dynamics. We observed fluctuations in community structure between samples, with depth (surface versus basal snow) and at temporal scales (taken over a 2-month period during the spring season). Since we did not replicate our metagenomic analysis within a same snow layer at the same time point, we cannot evaluate beta diversity and while we cannot exclude that these fluctuations reflect snowpack heterogeneity, microarray analysis presented in Larose et al. (2013c) suggested that modifications in environmental conditions may also be involved. For example, more constant conditions at the base of the snowpack might create more stable communities, including psychrotolerant/psychrophile organisms, to establish there. The high relative abundance of reads associated with *Fungi* in mid-May could be linked to spore deposition during snowfall and a fungal bloom due to snowpack wetting and warming. This heterogeneity in community structure between surface and basal snow due to variable conditions has been previously observed in Greenland snowpacks (Møller et al., 2013). Environmental chemistry, such as pH, organic acids, nitrogen, sulfur cycling, and Hg, has been correlated with bacterial community dynamics measured by 16S rRNA gene phylogenetic microarray in the Svalbard snowpack (Larose et al., 2013c). Unfortunately, taxonomical data associated with ribosomal gene analyses cannot provide the details of the ecological role of these organisms, since functional potential can differ between species and within the same taxonomic species (Polz et al., 2013). Functional metagenomic analysis has been proposed as a technique for assessing ecosystem ecology (e.g., how microorganisms are adapted to a given environment and what role they play in this environment (Dinsdale et al., 2008; Delmont et al., 2011a; Simon and Daniel, 2011).

The relative abundance of functionally unannotated reads was very high in our snow metagenomes, illustrating the lack of environmental representatives in databases, especially from largely unexplored environments such as snowpacks. However, our preliminary data indicate that the Arctic snowpack harbors a specific functional signature based on gene annotation of sequenced DNA and that this functional potential is correlated to the varying environmental conditions during the spring season and with depth in the snowpack. We observed a correlation between specific functional gene abundance and chemical parameters. For example, Hg and bio-Hg were correlated to the subsystem tetrapyrroles in which most of the reads are associated with heme and siroheme biosynthesis and cobalamin and coenzyme B12 biosynthesis. Cobalamin has been shown to be involved in Hg methylation in sulfate-reducing bacteria (Choi et al., 1994; Parks et al., 2013). This supports the hypothesis that snowpack is a dynamic ecosystem that responds to changes in environmental conditions (Larose et al., 2013c), but this functional exploration also uncovered some of the factors that might drive microbial community structure and function in the snowpack ecosystem. The Arctic snowpack is an important component of the cryosphere (Priscu and Christner, 2004) and shares many extreme environment characteristics with other frozen habitats, like sea ice and polar mats. These characteristics include low nutrient concentrations, desiccation due to low water activity, a freeze-thaw cycle, and intense UV irradiation during summer and darkness during winter as well as low temperatures (Priscu and Christner, 2004). Fungi using effective adaptation mechanisms might be able to grow and develop in such habitats and are not just windblown contaminant spores (Gunde-Cimerman et al., 2003). If this is the case, then *Fungi* might be carrying out several different metabolic activities in the snow. Unfortunately, the large majority of *Fungi* affiliated reads in our metagenomic datasets (up to 86%) were not functionally annotated due to a lack of fungal genomic and protein data, and therefore, we were unable to clearly characterize the functional potential of the fungal microbial community. Some bacterial adaptation mechanisms have been described for how microorganisms in culture deal with these cold habitats (Deming, 2002; Rodrigues and Tiedje, 2008). These consist of polyunsaturated fatty acid biosynthesis involved in maintenance of membrane fluidity, production of exopolysachharides, cold shock protein, DNA gyrase maintaining DNA topology or choline betain uptake. The vast amount of metagenomic sequence data could provide a rich source for the mining of psychrophilic adaptations from uncultured organisms (Casanueva et al., 2010). We observed sequence reads associated with the genes considered to be related to these adaptation mechanisms in our snow metagenomes. Similar observations were carried out in glacier ice and polar mat microbial community metagenomes (Simon et al., 2009; Varin et al., 2012b), pointing out the role of these mechanisms for adaption in cold environments. But cold adaptation does not only occur in extreme environments and we detected these functions in all other ecosystem metagenomes. This implies that other adaptive mechanisms related to environmental factors may play a role in defining the snow microbial community structure and function relative to other ecosystems. One such environmental factor might include constant light irradiation during the summer months in the Arctic. This intense irradiation has been described as playing a major role in snowpack chemistry due to photochemical reactions (including oxidative chemical reactions; Grannas et al., 2007). Constant light irradiation could also affect regulation of light-dependent metabolisms, especially in photosynthetic microorganisms. Cyanobacterial circadian clock functions appeared more dominant in our snow metagenomes (and in polar microbial mat; Varin et al., 2012a) than in other ecosystems. *Cyanobacteria* are the simplest organisms known to have endogenous circadian clock mechanisms (Kondo et al., 1993). The circadian input kinase (cikA) gene encodes a bacterio-phytochrome-like histidine kinase involved in the input signaling of the clock (Schmitz, 2000). These mechanisms might be crucial for light-dependent metabolisms under constant light conditions. Moreover, UV-light irradiation might be an especially important factor in microbial snow community ecology in arctic snowpacks. Described as a natural photochemical bioreactor (Grannas et al., 2007; Domine et al., 2008), snowpacks are highly reactive to UV light. Chemical compounds sequestered in snowpacks are photolyzed upon irradiation and reactive trace gases are released in the snow boundary layer. These photochemical-induced reactions may result in the accumulation of reactive species within the snowpack, and thus, in the emergence of a hyper oxidative stress habitat. Oxidative stress is defined by a physiological state produced when the concentration of reactive oxygen species exceeds cell defense capacity as expressed during aerobic metabolism. Free radicals are highly reactive chemical species that damage DNA, proteins or lipids via oxidation; lipid peroxidation, for example, causes cell membrane degradation (Cabiscol et al., 2000). This stress can be caused by the diffusion of radicals into the cell or by environmental agents, such as ions, near-UV radiation and other compounds that generate intracellular radicals (Cabiscol et al., 2000). Some functional subsystems associated with potential oxidative stress response, such as oxidative stress and NAD/NADP metabolism, were among the 50 most abundant subsystems in relative abundance of annotated reads. Moreover, the abundance of reads annotated within these oxidative stress subsystems showed a strong correlation with Hg, bio-Hg, and nitrite concentrations in our samples. During Hg exposure, oxygen reactive species like hydrogen peroxide might increase due to the direct effect of Hg^2+^ on electron transport pathways (Lund et al., 1993) and to the Hg^2+^-mediated activation of super oxide stress responses (Smith et al., 1998). Reactive nitrogen species (RNS), like nitric oxide, are formed as metabolic side products when nitrate or nitrite is used as a terminal electron acceptor in denitrifying bacteria (Goretski et al., 1990) or by the reduction of nitrite to NO^.^ by an oxidase (Bonner et al., 1991) and also generated exogenously by nitrate or nitrite photolysis (Grannas et al., 2007). These RNS might be involved in the induction of oxidative stress response mechanisms like hemoglobins (Frey et al., 2002).The NAD/NADP and quinine cofactors subsystem might be involved in oxidative stress response in the snowpack microbial community. NADH/NADPH pools represent essential non enzymatic antioxidants within bacterial cells (Cabiscol et al., 2000). In *Escherichia coli*, a fine regulation of NADP(H) homeostasis is necessary for proper deployment of the oxidative stress defensive response (Krapp et al., 2011). The widely distributed NAPH quinone reductase is implicated in the oxidative stress resistance during host colonization by *Helicobacter pylori* (Wang and Maier, 2004). Reads associated with Sigma B factor, required for the induction of approximately 100 genes responding to a whole range of stresses (Völker et al., 1999), were more abundant in our snow metagenomes than in other ecosystems. In particular, oxidative stress response is mediated by sigma factor in a wide range of microorganisms such as *Bacillus subtilis* (Völker et al., 1999), *Streptomyces coelicolor* (Paget et al., 1998), and *Listeria monocytogenes* (Ferreira et al., 2001). This function has also been identified in genera from nutritionally poor aquatic environments, such as *Caulobacter* (Alvarez-Martinez et al., 2007), which were detected in our snow metagenomes*.* Moreover, part of these oxidative stress response associated functions (i.e., bacterial hemoglobins, NAD/NADP or Sigma B stress response regulation) were more highly represented in snow metagenomes than in other ecosystems. Although polar microbial mats in ice shelves are also exposed to high UV irradiation, high photosynthetically active radiation and photodamage (Roos and Vincent, 1998; Mueller et al., 2005), the highly structured multilayer microbial community contains a high amount of pigments such as Scytonemin and its reduced derivatives. These pigments could act as an effective sunscreen protecting the entire community against UV exposure in ice shelf polar mats (Lionard et al., 2012). Given that the microorganisms in snowpacks are not structured with comparable complexity and thickness, they likely do not benefit from similar protective effects. The results presented here support the possibility that photochemistry, given the high light exposure in snow that may result in oxidative stress conditions, might be an important factor for defining microbial community structure in the Arctic spring snowpack. However, one of the microbial sources for snowpacks is the atmosphere and microorganisms in clouds in the atmosphere are also exposed to high UV irradiance and some present corresponding adaptations such as pigments (Delort et al., 2010). Cloud microflora exposed to UV light in microcosms also remained metabolically active in the presence of .OH radicals (Vaïtilingom et al., 2013). While our data does not allow us to clearly discriminate between adaptation to stresses encountered during atmospheric transport and those selected for after deposition in snowpack, snow and atmosphere were shown to present distinct microbial assemblages in other reports (Bowers et al., 2009; Harding et al., 2011). In addition, the rapid selection of microorganisms after deposition to constitute a snow specific microbial community has been suggested (Segawa et al., 2005) with, for example, some plant pathogens belonging to *Agrobacterium* that are likely wind transported and which are detected in fresh snow and not after the snowfall event (Larose et al., 2013c). Metagenomic comparison of different snow layers and the corresponding atmosphere samples will help to address how microorganisms are selected after deposition. In the same vein, metatranscriptomic analyses will also be useful to determine which part of this community is active at a given period and what their roles in the snow ecosystem are.

## CONCLUSION

This study explored the microbial community functional genes in the Arctic snowpack. This microbial community, including representative members associated with cold environments, underwent major changes during the Spring season. Functional data that correlated with chemical parameters supported the hypothesis that this variation in microbial community structure and function could be explained by fluctuations in environmental conditions. Moreover, in this study, we tested the occurrence of a specific functional signature from the snowpack microbial community. Intense UV-light irradiation might be a critical factor in defining the microbial ecology of the Arctic snowpack ecosystem. Further sampling during the dark period as well as metatranscriptomic and atmosphere comparison studies year round would help establish how microorganisms are selected in snowpack and the role of light as a major driver of snowpack microbial community structure and function.

## Conflict of Interest Statement

The authors declare that the research was conducted in the absence of any commercial or financial relationships that could be construed as a potential conflict of interest.
