# Supplementary material for: Potential drivers of microbial community structure and function in Arctic spring snow
Source: Front Microbiol. 2014 Aug 7;5:413. doi: 10.3389/fmicb.2014.00413 (PMC4124603; doi:10.3389/fmicb.2014.00413)
Supplement: Supplementary file 1 [file DataSheet_1.DOCX]

**Table S1:** Publically available metagenomic datasets used for environmental comparison, deposited in MG-RAST sofawre (Meyer et al. 2008)

| Metagenome | Environment | Metagenome Name | bp Count | Sequence Count | Biome | References |
| --- | --- | --- | --- | --- | --- | --- |
| 4451073.3 | snow | svn8 | 1.11E+07 | 2.91E+04 | Snow | This Study |
| 4451070.3 | snow | svn7 | 1.49E+07 | 4.30E+04 | Snow | This Study |
| 4451071.3 | snow | svn65 | 4.05E+06 | 1.22E+04 | Snow | This Study |
| 4451072.3 | snow | svn64 | 6.86E+06 | 2.16E+04 | Snow | This Study |
| 4451069.3 | snow | svn56 | 7.77E+06 | 2.55E+04 | Snow | This Study |
| 4451076.3 | snow | svn40 | 8.04E+06 | 2.37E+04 | Snow | This Study |
| 4451065.3 | snow | svn35 | 1.17E+07 | 3.48E+04 | Snow | This Study |
| 4451075.3 | snow | svn18 | 1.10E+07 | 3.84E+04 | Snow | This Study |
| 4451068.3 | snow | svn48 | 7.76E+06 | 2.43E+04 | Snow | This Study |
| 4446892.3 | soil | RothE1 | 3.84E+08 | 1.05E+06 | Grassland_Soil | (Delmont et al. 2011) |
| 4446896.3 | soil | RothE41 | 5.02E+08 | 1.23E+06 | Grassland_Soil | (Delmont et al. 2011) |
| 4446895.3 | soil | RothE4B | 3.62E+08 | 1.02E+06 | Grassland_Soil | (Delmont et al. 2011) |
| 4446894.3 | soil | RothE4A | 5.19E+08 | 1.22E+06 | Grassland_Soil | (Delmont et al. 2011) |
| 4446902.3 | soil | RothF47 | 5.51E+08 | 1.24E+06 | Grassland_Soil | (Delmont et al. 2011) |
| 4446903.3 | soil | RothF48 | 4.36E+08 | 1.06E+06 | Grassland_Soil | (Delmont et al. 2011) |
| 4446904.3 | soil | RothL01 | 3.50E+08 | 8.60E+05 | Grassland_Soil | (Delmont et al. 2011) |
| 4446153.3 | soil | Puerto_Rico_Soil | 3.22E+08 | 7.82E+05 | Forest_Soil | (DeAngelis et al. 2011) |
| 4443697.3 | open_ocean | open_ocean_P1 | 6.81E+07 | 2.93E+05 | Marine_Habitat | MgRast (Meyer et al. 2008) |
| 4443725.3 | open_ocean | open_ocean_NA1 | 5.90E+07 | 2.57E+05 | Marine_Habitat | MgRast (Meyer et al. 2008) |
| 4443729.3 | open_ocean | open_ocean_NA2 | 6.74E+07 | 2.89E+05 | Marine_Habitat | MgRast (Meyer et al. 2008) |
| 4443713.3 | coast_ocean | coast_ocean_MB1 | 5.30E+07 | 2.22E+05 | Marine_Habitat | MgRast (Meyer et al. 2008) |
| 4443714.3 | coast_ocean | coast_ocean_MB2 | 4.50E+07 | 1.89E+05 | Marine_Habitat | MgRast (Meyer et al. 2008) |
| 4443716.3 | coast_ocean | coast_ocean_MB3 | 5.30E+07 | 2.23E+05 | Marine_Habitat | MgRast (Meyer et al. 2008) |
| 4445126.3 | Polar_Microbial_Mat | Polar_Microbial_Mat_MIS | 5.34E+07 | 2.57E+05 | Polar Micorboial Mats | (Varin et al. 2012b) |
| 4445129.3 | Polar_Microbial_Mat | Polar_Microbial_Mat_WHI | 6.17E+07 | 3.36E+05 | Polar Micorboial Mats | (Varin et al. 2012b) |
| 4445845.3 | Polar_Microbial_Mat | Polar_Microbial_Mat_MM | 3.10E+07 | 8.33E+04 | Polar Micorboial Mats | (Varin et al. 2012b) |
