# Supplementary material for: Potential drivers of microbial community structure and function in Arctic spring snow
Source: Front Microbiol. 2014 Aug 7;5:413. doi: 10.3389/fmicb.2014.00413 (PMC4124603; doi:10.3389/fmicb.2014.00413)
Supplement: Supplementary file 2 [file DataSheet_2.DOCX]

**Table S2:** Functions more represented in snow and polar mat ecosystems than in other ecosystems (level 2 seed annotation)

| level 1 | level 2 | p-values | Polar Microbial Mat | coast ocean | open ocean | soil | snow |
| --- | --- | --- | --- | --- | --- | --- | --- |
| Regulation and Cell signaling | Two-component regulatory systems in Campylobacter | 2.77E-06 | 0.120 | 0.013 | 0.009 | 0.078 | 0.174 |
| Phages, Prophages, Transposable elements, Plasmids | Transposable elements | 8.33E-04 | 0.159 | 0.052 | 0.050 | 0.133 | 0.198 |
| Clustering-based subsystems | Biosynthesis of galactoglycans and related lipopolysacharides | 1.26E-03 | 0.606 | 0.161 | 0.211 | 0.538 | 0.762 |
| Regulation and Cell signaling | Regulation of virulence | 1.29E-03 | 0.188 | 0.182 | 0.146 | 0.150 | 0.307 |
| Cofactors, Vitamins, Prosthetic Groups, Pigments | NAD and NADP | 1.72E-03 | 0.507 | 0.424 | 0.467 | 0.490 | 0.745 |
| Iron acquisition and metabolism | Iron acquisition in Vibrio | 2.29E-03 | 0.279 | 0.294 | 0.229 | 0.191 | 0.679 |
| Membrane Transport | Transport of Manganese | 2.66E-03 | 0.074 | 0.041 | 0.035 | 0.080 | 0.089 |
| Clustering-based subsystems | CBSS-176280.1.peg.1561 | 5.00E-03 | 0.107 | 0.072 | 0.047 | 0.094 | 0.148 |
| Clustering-based subsystems | Shikimate kinase containing cluster | 1.41E-02 | 0.036 | 0.046 | 0.046 | 0.032 | 0.078 |
| Clustering-based subsystems | CBSS-235.1.peg.567 | 2.13E-02 | 0.303 | 0.314 | 0.270 | 0.215 | 0.331 |
| Clustering-based subsystems | CBSS-288681.3.peg.1039 | 2.57E-02 | 0.062 | 0.003 | 0.005 | 0.028 | 0.072 |
| Clustering-based subsystems | CBSS-316273.3.peg.2709 | 2.57E-02 | 0.062 | 0.003 | 0.005 | 0.028 | 0.072 |
| Carbohydrates | Monosaccharides | 2.62E-02 | 1.089 | 0.825 | 0.830 | 1.307 | 1.384 |
| Respiration | Soluble cytochromes and functionally related electron carriers | 3.28E-02 | 0.197 | 0.094 | 0.121 | 0.165 | 0.264 |
| Regulation and Cell signaling | Programmed Cell Death and Toxin-antitoxin Systems | 3.84E-02 | 0.107 | 0.015 | 0.013 | 0.067 | 0.157 |
| Carbohydrates | Aminosugars | 3.88E-02 | 0.172 | 0.103 | 0.090 | 0.180 | 0.239 |
| Clustering-based subsystems | Staphylococcus aureus hypothetical repetitive gene loci | 3.91E-02 | 0.069 | 0.051 | 0.053 | 0.067 | 0.106 |
| Potassium metabolism | Potassium homeostasis | 3.93E-02 | 0.257 | 0.182 | 0.137 | 0.373 | 0.403 |
| Fatty Acids, Lipids, and Isoprenoids | Phospholipids | 4.92E-02 | 0.475 | 0.413 | 0.437 | 0.454 | 0.579 |
| Carbohydrates | Glycoside hydrolases | 4.93E-02 | 0.036 | 0.014 | 0.011 | 0.043 | 0.065 |
| Stress Response | Bacterial hemoglobins | 5.64E-02 | 0.054 | 0.004 | 0.003 | 0.047 | 0.114 |
| Iron acquisition and metabolism | Transport of Iron | 6.61E-02 | 0.091 | 0.118 | 0.131 | 0.071 | 0.144 |
| Cell Wall and Capsule | Gram-Positive cell wall components | 7.18E-02 | 0.094 | 0.069 | 0.076 | 0.106 | 0.206 |
| Iron acquisition and metabolism | Iron(III) dicitrate transport system Fec | 7.49E-02 | 0.006 | 0.015 | 0.002 | 0.002 | 0.027 |
| Membrane Transport | Ton and Tol transport systems | 8.09E-02 | 0.364 | 0.381 | 0.283 | 0.307 | 0.633 |
| Stress Response | SigmaB stress responce regulation | 1.43E-01 | 0.046 | 0.004 | 0.010 | 0.044 | 0.053 |
| Clustering-based subsystems | Spore Coat | 1.44E-01 | 0.016 | 0.007 | 0.007 | 0.023 | 0.052 |
| Potassium metabolism | Glutathione-regulated potassium-efflux system and associated functions | 1.51E-01 | 0.054 | 0.029 | 0.024 | 0.030 | 0.067 |
| Sulfur Metabolism | Inorganic sulfur assimilation | 1.59E-01 | 0.384 | 0.270 | 0.259 | 0.364 | 0.430 |
| Cofactors, Vitamins, Prosthetic Groups, Pigments | Coenzyme M | 1.71E-01 | 0.008 | 0.014 | 0.015 | 0.011 | 0.025 |
| Regulation and Cell signaling | Proteolytic pathway | 1.85E-01 | 0.001 | 0.000 | 0.003 | 0.006 | 0.053 |
| Photosynthesis | Electron transport and photophosphorylation | 1.98E-01 | 0.244 | 0.030 | 0.199 | 0.002 | 0.306 |
| Clustering-based subsystems | CBSS-316273.3.peg.2378 | 1.45E-05 | 0.046 | 0.004 | 0.004 | 0.005 | 0.008 |
| DNA Metabolism | Type I Restriction-Modification | 9.56E-05 | 0.356 | 0.032 | 0.026 | 0.102 | 0.245 |
| DNA Metabolism | Restriction-Modification System | 1.11E-04 | 0.391 | 0.035 | 0.035 | 0.113 | 0.272 |
| Photosynthesis | Light-harvesting complexes | 4.38E-04 | 0.109 | 0.003 | 0.026 | 0.000 | 0.028 |
| Cell Division and Cell Cycle | Cyanobacterial Circadian Clock | 1.05E-03 | 0.129 | 0.003 | 0.037 | 0.032 | 0.127 |
| Clustering-based subsystems | CBSS-196620.1.peg.2477 | 1.33E-03 | 0.291 | 0.103 | 0.045 | 0.176 | 0.215 |
| Iron acquisition and metabolism | Campylobacter Iron Metabolism | 2.17E-02 | 0.121 | 0.109 | 0.099 | 0.062 | 0.115 |
| Nitrogen Metabolism | Nitrate and nitrite ammonification | 2.62E-02 | 0.180 | 0.022 | 0.014 | 0.135 | 0.162 |
| Clustering-based subsystems | LMPTP YfkJ cluster | 5.04E-02 | 0.042 | 0.011 | 0.005 | 0.035 | 0.040 |
| Stress Response | Acid stress | 5.72E-02 | 0.066 | 0.009 | 0.021 | 0.044 | 0.059 |
| Regulation and Cell signaling | Oxygen and light sensor PpaA-PpsR | 6.32E-02 | 0.059 | 0.006 | 0.008 | 0.019 | 0.040 |
| Amino Acids and Derivatives | Arginine; urea cycle, polyamines | 7.53E-02 | 1.537 | 0.973 | 1.055 | 1.219 | 1.308 |
| Clustering-based subsystems | CRISPRs and associated hypotheticals | 9.52E-02 | 0.021 | 0.000 | 0.000 | 0.008 | 0.012 |
| Clustering-based subsystems | CBSS-393131.3.peg.612 | 1.17E-01 | 0.026 | 0.005 | 0.003 | 0.013 | 0.022 |
| DNA Metabolism | CRISPs | 1.20E-01 | 0.051 | 0.001 | 0.001 | 0.020 | 0.039 |
| Clustering-based subsystems | Possible Ammonia conversion cluster | 1.39E-01 | 0.091 | 0.073 | 0.080 | 0.050 | 0.089 |
