# Supplementary material for: Potential drivers of microbial community structure and function in Arctic spring snow
Source: Front Microbiol. 2014 Aug 7;5:413. doi: 10.3389/fmicb.2014.00413 (PMC4124603; doi:10.3389/fmicb.2014.00413)
Supplement: Supplementary file 3 [file DataSheet_3.DOCX]

| Cold adptation related subsystems | p-values | Cryospheric: mean rel. freq. (%) | Cryospheric: std. dev. (%) | Mesophylic: mean rel. freq. (%) | Mesophylic: std. dev. (%) | Polar Microbial Mat: mean rel. freq. (%) | coast ocean: mean rel. freq. (%) | open ocean: mean rel. freq. (%) | snow: mean rel. freq. (%) | soil: mean rel. freq. (%) |
| --- | --- | --- | --- | --- | --- | --- | --- | --- | --- | --- |
| AceE (pyruvate dehydrogenase E1 component) | 1.01E-02 | 9.66 | 6.71 | 15.02 | 1.50 | 14.06 | 14.74 | 14.24 | 8.19 | 15.41 |
| AceF (dihydrolipoamide acetyltransferase) | 4.82E-01 | 6.95 | 4.70 | 5.97 | 1.38 | 6.25 | 5.72 | 4.44 | 7.19 | 6.64 |
| Chaperone DnaK and DnaJ | 3.16E-01 | 6.20 | 2.81 | 5.35 | 0.86 | 7.68 | 4.24 | 6.09 | 5.70 | 5.50 |
| Choline and betaine uptake, betaine biosynthesis | 9.16E-01 | 8.57 | 16.92 | 8.05 | 4.59 | 3.34 | 13.93 | 12.47 | 10.31 | 4.19 |
| DnaA (replication initiator protein) | 6.97E-01 | 3.97 | 2.86 | 4.30 | 0.71 | 3.60 | 4.44 | 3.58 | 4.09 | 4.51 |
| Exopolysaccharide biosynthesis | 3.21E-01 | 0.73 | 2.20 | 0.11 | 0.11 | 0.24 | 0.00 | 0.12 | 0.89 | 0.14 |
| Fatty acid desaturases | 4.66E-02 | 3.34 | 2.56 | 1.78 | 0.71 | 2.87 | 0.85 | 1.15 | 3.50 | 2.37 |
| Glutamate biosynthesis | 2.62E-01 | 24.28 | 10.10 | 27.67 | 3.00 | 26.81 | 23.68 | 26.83 | 23.44 | 29.48 |
| Glycine biosynthesis | 8.93E-02 | 7.02 | 4.29 | 4.87 | 0.79 | 4.12 | 4.84 | 3.66 | 7.99 | 5.34 |
| GyrA (DNA gyrase A) | 3.72E-01 | 9.63 | 7.63 | 11.60 | 1.46 | 14.66 | 12.38 | 12.92 | 7.96 | 10.82 |
| HU-β (DNA supercoiling) | 2.48E-01 | 0.55 | 0.96 | 0.23 | 0.14 | 0.18 | 0.16 | 0.33 | 0.68 | 0.22 |
| OstA (trehalose phosphate synthase) | 4.31E-01 | 0.68 | 0.88 | 0.94 | 0.74 | 0.83 | 0.05 | 0.23 | 0.62 | 1.54 |
| Peptidyl-prolyl cis-trans isomerase | 2.15E-01 | 0.85 | 0.94 | 0.51 | 0.22 | 1.17 | 0.45 | 0.34 | 0.74 | 0.59 |
| Purine nucleoside phosphorylase (PNP) | 1.11E-01 | 1.34 | 1.74 | 0.53 | 0.24 | 0.68 | 0.57 | 0.16 | 1.56 | 0.65 |
| RecA (recombination factor A) | 6.57E-01 | 12.98 | 13.13 | 11.33 | 1.47 | 10.36 | 11.64 | 11.78 | 13.86 | 11.05 |
| tRNA dihydrouridine synthase | 6.01E-02 | 3.24 | 2.69 | 1.74 | 0.47 | 3.14 | 2.31 | 1.66 | 3.28 | 1.55 |

**Table S3:** Relative abundance of annotated reads among known cold adaptation related mechanisms. P-values are calculated with Anova analysis between cryospheric environments (Polar Microbial Mat and Snow) and mesophilic (Soil, Open ocean and Coastal ocean).
